# Artichoke, Cynarin and Cyanidin Downregulate the Expression of Inducible Nitric Oxide Synthase in Human Coronary Smooth Muscle Cells

**DOI:** 10.3390/molecules19033654

**Published:** 2014-03-24

**Authors:** Ning Xia, Andrea Pautz, Ursula Wollscheid, Gisela Reifenberg, Ulrich Förstermann, Huige Li

**Affiliations:** Department of Pharmacology, Johannes Gutenberg University Medical Center, Obere Zahlbacher Str. 67, 55131 Mainz, Germany

**Keywords:** nitric oxide, inducible NO synthase, vascular smooth muscle cells, artichoke, *Cynara scolymus* L.

## Abstract

Artichoke (*Cynara scolymus* L.) is one of the world’s oldest medicinal plants with multiple health benefits. We have previously shown that artichoke leaf extracts and artichoke flavonoids upregulate the gene expression of endothelial-type nitric oxide synthase (eNOS) in human endothelial cells. Whereas NO produced by the eNOS is a vasoprotective molecule, NO derived from the inducible iNOS plays a pro-inflammatory role in the vasculature. The present study was aimed to investigate the effects of artichoke on iNOS expression in human coronary artery smooth muscle cells (HCASMC). Incubation of HCASMC with a cytokine mixture led to an induction of iNOS mRNA expression. This iNOS induction was concentration- and time-dependently inhibited by an artichoke leaf extract (1–100 µg/mL, 6 h or 24 h). Consistently, the artichoke leaf extract also reduced cytokine-induced iNOS promoter activation and iNOS protein expression. In addition, treatment of HCASMC with four well-known artichoke compounds (cynarin > cyanidin > luteolin ≈ cynaroside) led to a downregulation iNOS mRNA and protein expression, with cynarin being the most potent one. In conclusion, artichoke contains both eNOS-upregulating and iNOS-downregulating compounds. Such compounds may contribute to the beneficial effects of artichoke and may *per se* have therapeutic potentials.

## 1. Introduction

In blood vessels, nitric oxide (NO) can be produced by the endothelial-type NO synthase (eNOS) or the inducible NO synthase (iNOS). Under physiological conditions, iNOS is absent in the vasculature and vascular NO is mainly produced by eNOS. This enzyme is constitutively expressed in the endothelium and is activated by shear stress of the flowing blood or by agonists such as bradykinin and acetylcholine. NO produced in endothelial cells can diffuse into the underlying smooth muscle cells and induce vasodilation by stimulating NO-sensitive guanylyl cyclase. Endothelial NO can also diffuse into the blood and inhibit platelet aggregation and adhesion. In addition to these antihypertensive and antithrombotic actions, eNOS-derived NO also possesses multiple anti-atherosclerotic properties, including prevention of leukocyte adhesion to vascular endothelium and leukocyte migration into the vascular wall, inhibition of low-density lipoprotein oxidation, and inhibition of vascular smooth muscle cell proliferation [1,2]. Genetic depletion of eNOS leads to exacerbation of diet-induced atherosclerosis in the apolipoprotein E-knockout mouse model. The blood pressure of eNOS knockout mice is about 30% higher than that of wild-type animals [3].

Under conditions of inflammation, sepsis, or oxidative stress, iNOS expression can be induced in blood vessels. In contrast to the regulated production of NO by eNOS, iNOS may generate large amounts of NO over long periods of time, if substrate and cofactors are not limited. This excessive NO from iNOS leads to vascular dysfunction evident as impairment of both vasoconstriction and endothelium-dependent vasorelaxation. Several mechanisms have been proposed by which iNOS impairs contractile responses, including continuous activation of the soluble guanylyl cyclase [4]; abnormal vascular calcium regulation [5]; and oxidative modification of catecholamines [6]. In parallel, the endothelium-dependent, NO-mediated vasodilation response (e.g., to acetylcholine or bradykinin) is also impaired by iNOS. This may result from reduced NO production by eNOS [4,7] or enhanced inactivation of eNOS-derivied NO by superoxide [8]. Tetrahydrobiopterin is an essential cofactor for NO production by NOS enzymes. iNOS expressed in the endothelium competes with eNOS for tetrahydrobiopterin and reduces NO production from eNOS by limiting availability of tetrahydrobiopterin for eNOS [4]. The continuous generation of NO by iNOS induced in the media can impair the signal transduction cascade that links activation of endothelial receptors to the calcium-calmodulin-dependent activation of eNOS [7]. Moreover, the reduction of endothelium-dependent relaxation may be mediated in part by reduced reactivity of smooth muscle cells to NO [9]. 

The consequence of this dysregulation (impaired vasomotor reactivity to both vasoconstrictor and vasodilator agonists) can be seen, for example, in septic shock. Septic shock is characterized by massive arteriolar vasodilatation, hypotension, and microvascular damage. Inappropriate vasodilation, abnormal regulation of blood flow to organs, myocardial suppression, and interference with cellular respiration all contribute to hypotension and mortality in septic shock [3,10]. Bacterial endotoxins usually initiate the symptoms and the fall in blood pressure is predominantly due to excess NO production by iNOS induced in the vascular wall. Endotoxin administration in experimental animals leads to high expression of iNOS in vascular smooth muscle cells and impairs contractile responses [11]. Inhibitors of iNOS largely restore the contractile responses to agonists in animal models of sepsis [12] and reverse the hypotension of patients in septic shock [13]. iNOS mutant mice have a blunted hypotensive response to sepsis [14]. Recently, a non-synonymous SNP in the iNOS gene has been shown to be associated with increased susceptibility to septic shock in Chinese populations [15]. 

The induction of iNOS in the vasculature is also associated with enhanced formation of peroxynitrite [8,16,17], a key pathogenic mechanism in conditions such as septic shock, stroke, myocardial infarction, chronic heart failure, diabetes, and atherosclerosis [18,19]. iNOS is present in human atherosclerosis plaque. Genetic deficiency of iNOS reduces atherosclerosis in apolipoprotein E-knockout mice [20]. iNOS also contributes to tissue damage after cerebral ischemia. Inhibition of iNOS by selective pharmacologic inhibitors [21], or gene deletion of iNOS [22] reduces brain damage.

Collectively, eNOS and iNOS have opposite roles in the vasculature with eNOS being protective and iNOS mostly detrimental. Compounds that upregulate eNOS or downregulate iNOS are of therapeutic interest. In a previous study, we have demonstrated that artichoke leaf extracts and artichoke flavonoids enhance eNOS expression and NO production in human endothelial cells [23]. The present study shows that artichoke inhibits iNOS expression in vascular smooth muscle cells.

## 2. Results and Discussion

In the present study, we demonstrate for the first time that artichoke leaf extract and artichoke compounds downregulate iNOS expression human coronary artery smooth muscle cells when administered concurrently with an inflammatory stimulus. 

### 2.1. Artichoke Leaf Extracts Downregulate iNOS Expression

**Figure 1:**
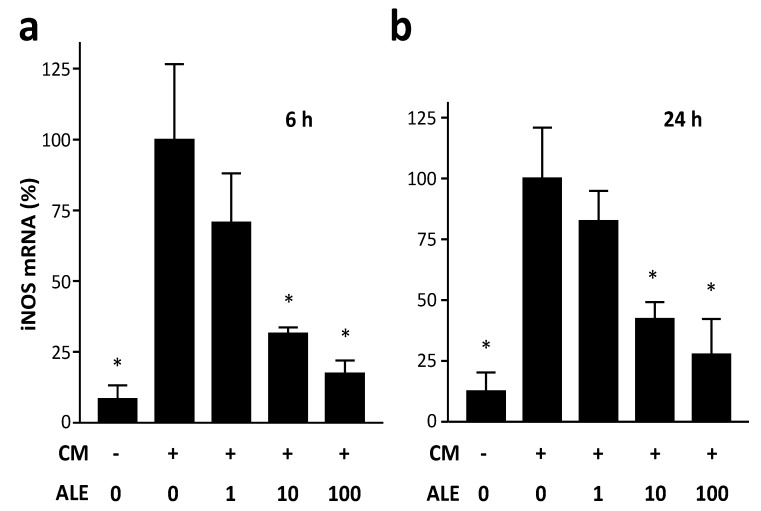
Artichoke leaf extracts downregulate iNOS mRNA expression. Human coronary artery smooth muscle cells were treated with the cytokine mixture (CM) or CM in combination with the artichoke leaf extract (ALE, µg/mL) for 6 h (**a**) or 24 h (**b**). Human iNOS mRNA expression was analyzed with real-time RT-PCR. * *p* < 0.05, compared with CM.

**Figure 2:**
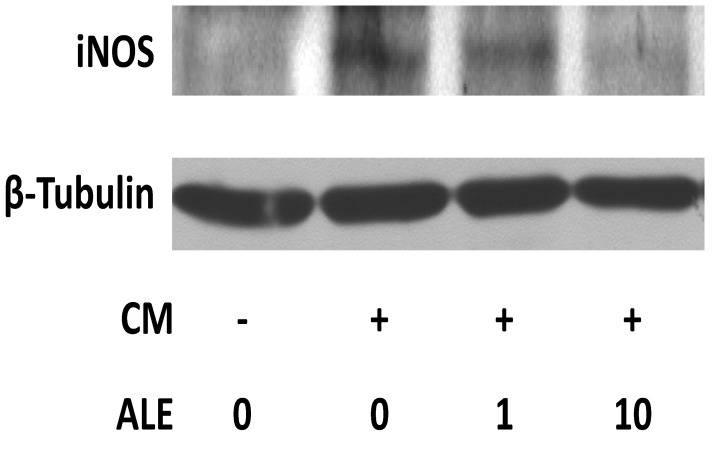
Artichoke leaf extracts downregulate iNOS protein expression. Human coronary artery smooth muscle cells were treated with the cytokine mixture (CM) or CM in combination with an artichoke leaf extract (ALE, µg/mL) for 24 h. Western blot analyses were performed with a monoclonal anti-iNOS-antibody (top) or a monoclonal antibody to β-tubulin (bottom, for normalization). The blots shown are representative of three independent experiments with similar results.

Incubation of HCASMC with the cytokine mixture (CM) led to an induction of iNOS mRNA expression. The induction of iNOS mRNA expression was more pronounced at 6 h than at 24 h (Figure 1). At both time points, the artichoke leaf extract (ALE) decreased the CM-induced iNOS expression at concentrations of 10 and 100 µg/mL. The kinetics of iNOS induction in HCASMC is likely to be similar as that in human intestinal epithelial DLD-1 cells in which the maximum of the CM-induced iNOS mRNA expression is reached at 6–12 h whereas iNOS protein expression and NO production are induced with a delay of several h [24]. Therefore, in the following experiments, iNOS mRNA expression and iNOS promoter activity were analyzed at 6 h whereas iNOS protein expression and NO production were studied at 24 h after CM treatment. At the protein level, ALE also concentration-dependently decreased the CM-induced iNOS expression (Figure 2). 

### 2.2. Artichoke Leaf Extracts Reduce Cytokine-Induced NO Production

Among the three NOS isoforms, iNOS is a calcium-independent enzyme. NO production by iNOS correlates well with the expression level of the enzyme. Therefore, the inhibition of iNOS expression by ALE should result in a reduction of NO production. 

**Figure 3:**
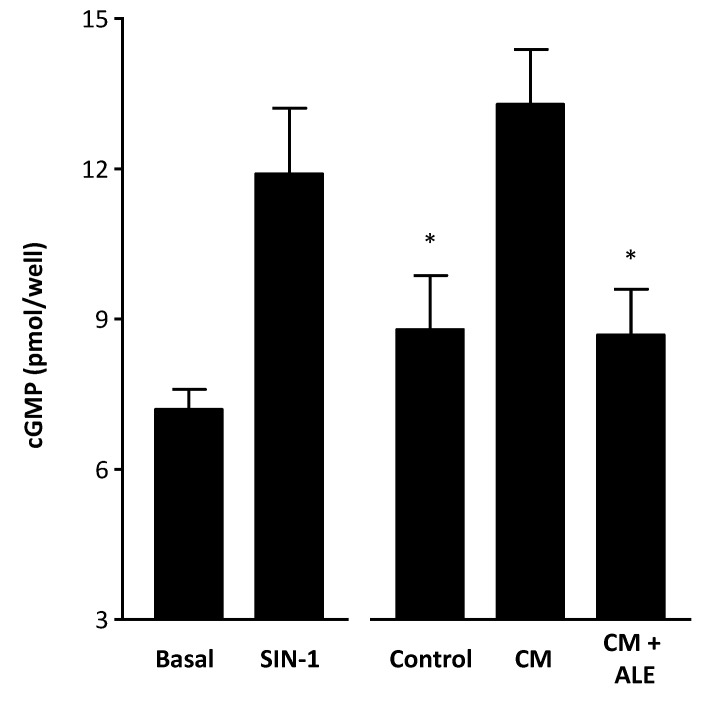
Artichoke leaf extracts prevent CM-induced cGMP production. Human coronary artery smooth muscle cells (HCASMC) were treated with the cytokine mixture (CM) or CM in combination with the artichoke leaf extract (ALE, 10 µg/mL) for 24 h. Then, conditioned media from HCASMC was transferred to RFL-6 reporter cells. The cGMP content in the RFL-6 cells was measured by radioimmunoassay. RFL-6 cells without conditioned media from HCASMC (basal) and RFL-6 cells treated for 3 min with the NO donor linsidomine (SIN-1, 1 µM) served as negative and positive controls, respectively. * *p* < 0.05, compared with CM.

To measure NO production from HCASMC, we applied the RFL-6 reporter cell assay. These cells express relatively high level of guanylyl cyclase and produce cGMP when stimulated with NO [25]. Incubation of RFL-6 cells with conditioned media from CM-treated HCASMC led to an increase of cGMP content, a surrogate marker for NO. The increase in cGMP content was significantly reduced by ALE (Figure 3).

### 2.3. Artichoke Leaf Extracts Inhibit iNOS Promoter Activity

The mRNA expression of iNOS can be regulated at the transcriptional level or the post-transcriptional level (via regulation of iNOS mRNA stability) [26]. We used a stably transfected cell line as a reliable and feasible tool for studying iNOS promoter activity. The stable A549/8 cells contain a 16 kb fragment of the human iNOS promoter cloned in front of a luciferase reporter gene [27]. The luciferase activity can be used as a measure of iNOS promoter activity. 

**Figure 4:**
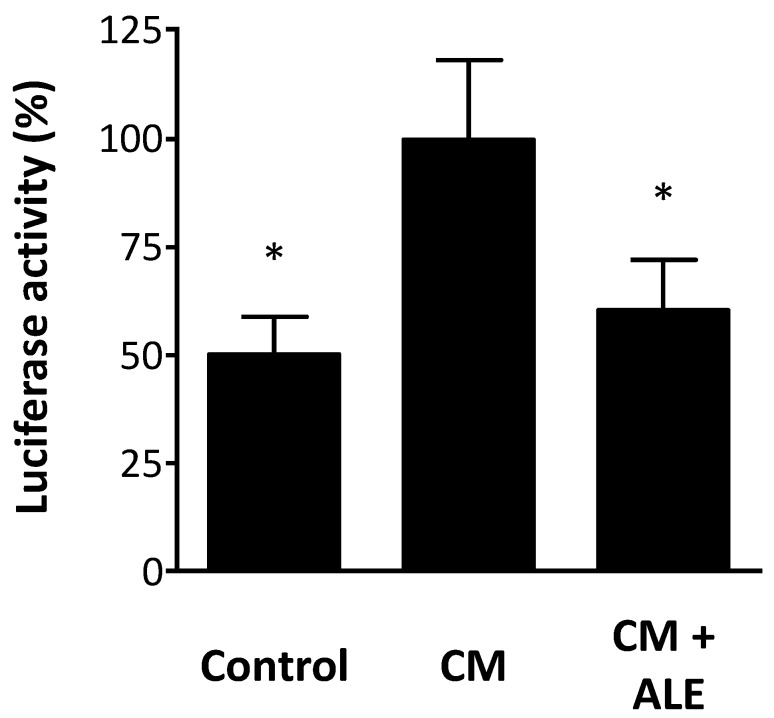
Artichoke leaf extracts reduce iNOS promoter activity. Human alveolar epithelium-like A549/8 cells were stably transfected with a construct containing a 16 kb fragment of the human iNOS promoter cloned in front of a luciferase reporter gene. The cells were treated with the cytokine mixture (CM) or CM in combination with an artichoke leaf extract (ALE, 10 µg/mL) for 6 h. Then, the cells were lysed, and luciferase activity was determined. The luciferase activity (normalized to protein content) was taken as a measure of iNOS promoter activity. * *p* < 0.05, compared with CM.

Treatment of the stable A549/8 cells with CM led to an induction of iNOS promoter activity (Figure 4). This transcriptional activation was significantly inhibited by ALE (Figure 4). Thus, the reduction of iNOS expression at mRNA and protein levels by ALE is likely to result, at least in part, from an inhibition of iNOS transcription. 

Cytokine-induced iNOS expression results from both transcriptional activation and post-transcriptional mechanisms (mRNA stabilization) [24]. This may be the reason why the induction of promoter activity (Figure 4) is less pronounced than that of mRNA expression (Figure 1). It is possible that ALE may also reduce iNOS expression partially by decreasing iNOS mRNA half-life. However, this remains an assumption at this time point as we did not study the effect of ALE on iNOS mRNA stability in the present study. 

### 2.4. Artichoke Compounds Inhibit iNOS Expression

Artichoke is rich in polyphenolic compounds, with mono- and dicaffeoylquinic acids as the major chemical components [28]. The most well-known caffeoylquinic acid derivative identified in artichoke extracts (heads and leaves), even though it is not the most abundant, is cynarin [28]. Besides caffeoylquinic acid derivatives, other phenolics belonging to the flavonoid class such as the flavones (e.g., luteolin and its 7-*O*-glucoside, cynaroside) and the anthocyanidins (e.g., cyanidin) have been identified in artichoke tissues [28]. Therefore, we studied the effects of these four well-known artichoke compounds on iNOS expression in HCASMC. 

**Figure 5:**
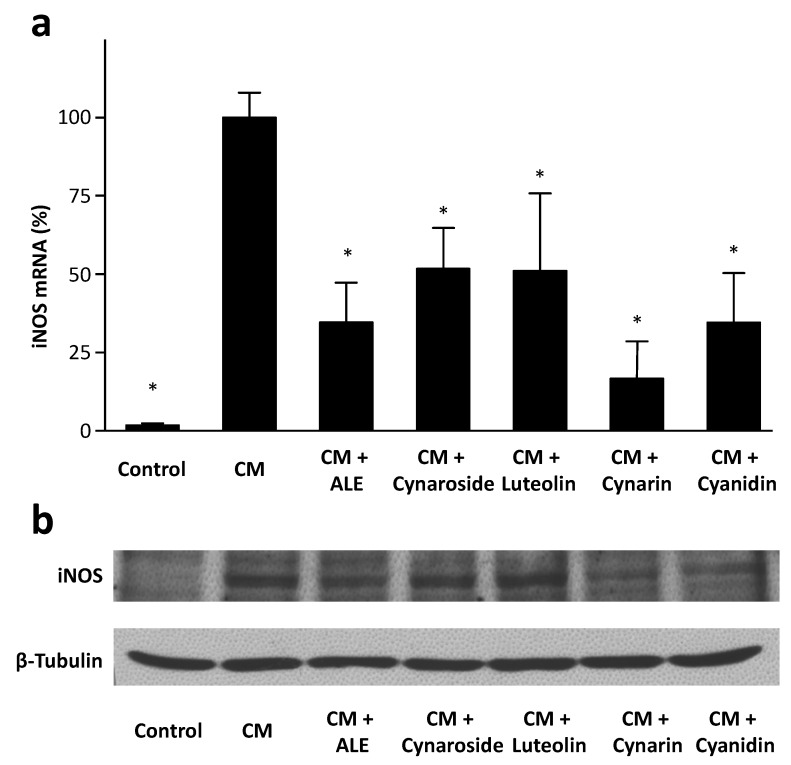
Artichoke compounds downregulate iNOS expression. Human coronary artery smooth muscle cells were treated with the cytokine mixture (CM) or CM in combination with an artichoke leaf extract (ALE, 10 µg/mL) or the artichoke compounds (10 µM each) for 6 h (**a**) or 24 h (**b**). Human iNOS mRNA expression was analyzed with real-time RT-PCR (**a**). * *p* < 0.05, compared with CM. Protein expression of iNOS was studied with Western blot analyses (**b**). The blots shown are representative of three independent experiments with similar results.

As shown in Figure 5, all four tested compounds (cynarin > cyanidin > luteolin ≈ cynaroside) downregulated iNOS mRNA expression in HCASMC, with cynarin being the most potent one (Figure 5a). At the protein level, cynarin and cyanidin clearly reduced iNOS expression, whereas luteolin and cynaroside had only minor effects (Figure 5b).

In our previous study, we have found that the flavone luteolin and its 7-*O*-glucoside cynaroside enhance eNOS expression in endothelial cells whereas the caffeoylquinic acids (cynarin and chlorogenic acid) are without effect [23]. These results, together with the data from the present study, indicate that the active constituents responsible for the artichoke effects on eNOS and iNOS are likely to be different compounds. 

### 2.5. Potential Therapeutic Relevance

Artichoke is one of the world’s oldest medicinal plants. It has been known by the ancient Egyptians, and the ancient Greeks and Romans used it as a digestive aid. Long known as an herbal medicine, the dried leaves of artichoke have been used in folk medicine because of their choleretic and hepatoprotective activities [28]. 

In various pharmacological test systems, artichoke leaf extracts have shown choleretic activity [29], hepatoprotective [30,31] and prebiotic effects [32,33]. In addition, artichoke leaf extracts contain compounds with antifungal [32] and antimicrobial activities [34]. 

Recent studies indicate that artichoke leaf extracts may also have therapeutic potential for cardiovascular disease. The plant contains multiple compounds with high antioxidant properties [35,36]. Antioxidative effects of artichoke extracts have been shown in endothelial cells [37,38] and leukocytes [37,39] *in vitro*, as well as in experimental animals *in vivo* [40,41]. Luteolin-rich artichoke extracts and luteolin itself protect low-density lipoprotein (LDL) from oxidation *in vitro* [42]. In a rat model of streptozotocin-induced diabetes, an artichoke leaf extract reduced the plasma malondialdehyde and urinary 8-hydroxydeoxyguanosine levels, and increased erythrocyte glutathione levels [41].

Although it is still a matter of debate [43], there is some evidence that artichoke leaf extract may lower cholesterol levels. In randomized, double-blind, placebo-controlled clinical trials, artichoke leaf extract reduced the total cholesterol levels in hypercholesterolemic adults [44,45].

A previous study from our laboratory shows that artichoke leaf extracts upregulate eNOS expression in human endothelial cells [23]. Increased NO production by artichoke extracts has also been shown in porcine aortic endothelial cells [46]. In a randomized, placebo-controlled trial, concentrated artichoke leaf juice significantly lowered blood pressure (by ≈ 3 mmHg after 12 weeks) in patients with mild hypertension [47]. 

The present study demonstrates that ALE, cynarin and cyanidin inhibit iNOS expression in vascular smooth muscle cells when administered concurrently with an inflammatory stimulus. We did not analyze whether the compounds in ALE have any effect on iNOS expression without an inflammatory stimulus. The cynarin concentration in the ALE we used is not known; but it could be in similar ranges as that in methanolic extracts of artichoke (≈1.5%) [28]. Based on this assumption, the cynarin concentration of 100 µg/mL ALE would be ≈3 µM, a concentration that is relevant to the effect of cynarin on iNOS expression. We are aware that we have only studied a few candidate compounds in the present study, and we may have missed a number of active compounds. It should also be reminded that the effects of artichoke extracts cannot be attributed to one or two single compounds. Rather, the *in vivo* effect of a plant extract is more likely to result from multiple active compounds that act additively or synergistically. It is also possible that some compounds contained in ALE may have antagonistic effects, or complex and unpredictable effects worthy of future study. 

The therapeutic potential of cynarin needs to be further investigated. Several issues should be considered in future studies including the bioavailability of the compound and the specificity of its action. After ingestion of artichoke extracts, cynarin and other caffeoylquinic acids are not found in human plasma. However, caffeoylquinic acid metabolites (such as caffeic acid, ferulic acid, isoferulic acid, dihydrocaffeic acid, and dihydroferulic acid) are detected in considerable concentrations [48,49]. It is still unknown whether the metabolites of cynarin are effective in inhibiting iNOS expression. In addition to its effect on iNOS, cynarin has antioxidative activities [39] and immuno-suppressive effects [50]. Recent studies indicate that cynarin may have the potential to serve as a chemosensitizing agent by reversing P-glycoprotein-mediated multidrug resistance [31]; but it may also induce drug-drug interactions by inhibiting organic anion transporters [51]. Therefore, animal studies are required to test the therapeutic potential and possible side effects of cynarin *in vivo*.

Anthocyanidins (e.g., cyanidin) and their glycosides (*i.e.*, anthocyanins) are responsible for the brilliant color of fruits and flowers and are widely ingested by humans [52]. Cyanidin and its glycosides show antioxidant, anti-inflammatory, and antimutagenic effects [52]. Cyanidin 3-*O*-β-d-glucoside reduces cytokine-induced iNOS and cyclooxygenase-2 (COX-2) expression in intestinal cells [53] and macrophages [54]. Moreover, cyanidin 3-*O*-β-d-glucoside has been shown to reduce ochratoxin-induced iNOS expression *in vivo* [55]. Orally administered cyanidin 3-*O*-β-d-glucoside is metabolized to cyanidin and protocatechuic acid by intestinal microflora [7] and these metabolites are detectable in blood and urine [56]. Cyanidin itself has also been shown to suppresses phorbol ester-induced COX-2 and iNOS expression in human colon adenocarcinoma cell line HT-29 cells [57] and in RAW 264.7 macrophages [56]. In an air pouch model of inflammation in mouse skin, both cyanidin 3-*O*-β-d-glucoside and cyanidin inhibit carrageenan-induced iNOS and COX2 expression, as well as the production of inflammatory cytokines after oral application [56]. These results are compatible with the present study and support the concept that cyanidin may have therapeutic potentials. 

## 3. Experimental

### 3.1. Cell Culture

Primary human coronary artery smooth muscle cells (HCASMC) were purchased from PromoCell (Heidelberg, Germany) and cultured in Smooth Muscle Cell Growth Medium 2 (PromoCell). For iNOS induction, HCASMC were incubated with a triple cytokine mixture (CM) containing IFN-γ (100 U/mL), IL-1β (50 U/mL) and TNF-α (10 ng/mL) [58]. To study the effects of artichoke, the cells were treated with CM in combination with LI-220 or artichoke compounds. LI-220 was an aqueous artichoke leaf extract (ALE) provided by Lichtwer Pharma AG (Berlin, Germany). Cynarin(1,5-dicaffeoylquinic acid; CAS number 30964-13-7) and cynaroside (luteolin-7-*O*-glucoside; CAS number 68321-11-9) were obtained from AppliChem (Darmstadt, Germany). Luteolin (CAS number 491-70-3) was from Calbiochem/Merck Millipore (Darmstadt, Germany) and cyanidin chloride (CAS number 528-58-5) was from Carl Roth (Karlsruhe, Germany). 

### 3.2. Real-Time RT-PCR for iNOS mRNA Analyses

Human iNOS mRNA expression was analyzed with quantitative real-time RT-PCR using an iCycler iQ System (Bio-Rad, Munich, Germany). Total RNA was isolated from HCASMC by guanidinium thiocyanatephenol-chloroform extraction. Total RNA (0.5 µg) was used for real-time RT-PCR analysis with the QuantiTect Probe RT-PCR kit (QIAGEN, Hilden, Germany). For real-time PCR, the following oligonucleotides served as sense and antisense primers and Taqman hybridization probes: iNOS, sense 5'- TGC AGA CAC GTG CGT TAC TCC-3', antisense 5'- GGT AGC CAG CAT AGC GGA TG-3', probe 5'-TGG CAA GCA CGA CTT CCG GGT G-3'; Pol2a (the large subunit of RNA polymerase II), sense 5'- GCA CCA CGT CCA ATG ACA T-3', antisense 5'-GTG CGG CTG CTT CCA TAA-3', probe 5'-TAC CAC GTC ATC TCC TTT GAT GGC TCC TAT-3' [58]. Pol2a was detected for normalization. 

### 3.3. Western Blot for iNOS Protein Analyses

Western blot analyses were performed with total protein samples (30 µg each) from HCASMC. Protein samples were separated on a Bis-Tris gel and transferred to a nitrocellulose membrane. Blots were blocked in 5% milk powder in TBST (10 mM Tris-HCl, pH 7.4, 150 mM NaCl with 0.1% Tween 20) for one h at room temperature. The primary antibody (a monoclonal anti-iNOS-antibody, R&D Systems, Wiesbaden, Germany) was diluted in the same solution used for blocking at 4 °C overnight. Blots were then washed in TBST and incubated with a horseradish peroxidase-conjugated secondary antibody diluted in 5% milk in TBST for one h. After washing in TBST and then in TBS, the immunocomplexes were developed using an enhanced horseradish peroxidase/luminol chemiluminescence reagent (PerkinElmer Life and Analytical Sciences, Boston, MA, USA) according to the manufacturer’s instructions [59]. 

### 3.4. Reporter Cell Assay for Determination of NO Production

NO production by HCASMC was bioassayed with RFL-6 rat lung fibroblasts as reporter cells [25,60]. HCASMC were treated with CM (with or without ALE) for 24 h. For determination of NO production, HCASMC and RFL-6 cells were washed twice with Locke’s solution (154.0 mM NaCl, 5.6 mM KCl, 2.0 mM CaCl_2_, 1 mM MgCl_2_, 3.6 mM NaHCO_3_, 5.6 mM glucose, 10.0 mM HEPES, pH 7.4). Then, HCASMC were incubated with Locke’s solution containing 200 U/mL SOD and 100 mM L-arginine, and RFL-6 cells with Locke’s solution containing 0.6 mM of the phosphodiesterase inhibitor IBMX for 20 min. After the preincubation, HCASMC were incubated for 2 min at 37 °C in 1 mL of Locke’s solution containing 200 U/mL SOD, 0.3 mM IBMX and 100 mM L-arginine. Then, the conditioned media containing the NO released from HCASMC were transferred to the RFL-6 cells, and another incubation of 2 min at 37 °C was performed. The reaction was stopped by aspiration of the solution, adding 1 mL of ice-cold 50 mM sodium acetate, pH 4.0, and rapidly freezing the cells with liquid nitrogen. The cGMP content of the RFL-6 samples was determined by radioimmunoassay as described [25,60].

### 3.5. Reporter Gene Assay for Determination of iNOS Promoter Activity

The human alveolar epithelium-like A549/8 cells were stably transfected with a construct containing a 16 kb fragment of the human iNOS promoter cloned in front of a luciferase reporter gene [27]. This cell line was used as a tool for analyzing iNOS promoter activity. The cells were treated with CM in the presence or absence of ALE. Then, cells were lysed in 1 × Passive Lysis Buffer (Promega). Firefly luciferase activity was determined using the Dual Luciferase Assay Kit (Promega). Protein concentrations of the extracts were determined by Bradford reagent using BSA as standard. Luciferase activity normalized to protein content of the extracts was used as a determinant of iNOS promoter activity [27].

### 3.6. Statistics

Data are presented as means with the standard deviation (SD). Statistical analyses were performed by one-way ANOVA with Bonferroni post-test. Differences were considered as significant when *p* < 0.05.

## 4. Conclusions

Artichoke is a medicinal plant with multiple health benefits. In the present study, we have found a new effect of artichoke leaf extracts and artichoke compounds (cynarin and cyanidin): inhibition of iNOS expression in vascular smooth muscle cells. The therapeutic potential of cynarin and cyanidin, however, needs to be verified in future studies *in vivo.*
